# The power of positivity: Exploring affective and social processes of change throughout a positive valence treatment for anxiety or depression^☆^

**DOI:** 10.1016/j.xjmad.2025.100146

**Published:** 2025-08-27

**Authors:** Madeleine Rassaby, Samantha N. Adams, Murray B. Stein, Charles T. Taylor

**Affiliations:** a San Diego State University/UC San Diego Joint Doctoral Program in Clinical Psychology, USA; b University of California San Diego, USA

**Keywords:** Anxiety, Depression, Positive affect, Negative affect, Social connectedness, Loneliness

## Abstract

**Objective:**

Social disconnection is common and disabling in persons with anxiety or depression, yet remains underexplored in treatment research. Proposed mechanisms underlying social disconnection include heightened negative valence system activation (increased negative affect [NA] and sensitivity to social threats) and diminished positive valence system activation (reduced positive affect [PA] and sensitivity to social rewards). Better understanding whether and how changes in affect relate to social connectedness throughout treatment may inform targets for remediation. This study addressed this question among individuals with clinically significant anxiety or depression and impaired social connectedness and functioning who completed a positive valence-targeted psychosocial intervention, Amplification of Positivity (AMP).

**Method:**

A secondary analysis was conducted using data from two trials (NCT03196544; NCT049452390), comprising 98 participants randomized to AMP. Longitudinal cross-lagged panel models, accounting for autoregression, examined the within-person, time-dependent relationships between PA and NA (entered concurrently) and subsequent social connectedness (loneliness and friendship) throughout treatment (Aim 1). Reverse models examined the relationships between connectedness and subsequent PA and NA (Aim 2). An exploratory analysis substituted anxiety and depressive symptoms for NA.

**Results:**

Higher PA predicted greater perceptions of friendship and lower loneliness at the following time point; NA was not significantly related to either outcome when examined concurrently with PA. Greater perceived friendship predicted higher subsequent PA; the effect of loneliness was non-significant. Neither friendship nor loneliness levels predicted subsequent NA. The same patterns were observed in exploratory symptom models, except lower anxiety (as well as higher PA) predicted lower subsequent loneliness.

**Conclusions:**

Higher PA (but not NA) predicted greater subsequent perceived friendship and lower loneliness throughout treatment.

## Highlights

• Examined temporal link between affect and connectedness in treatment for anxiety or depression.
• Higher PA (but not NA) predicted greater subsequent perceived friendship and lower loneliness.
• Greater friendship (but not loneliness) predicted higher subsequent PA.
• Neither friendship nor loneliness levels predicted subsequent NA.

## 1 Introduction

### 1.1 Social disconnection in anxiety and depression

Social disconnection is a persistent and impairing issue commonly experienced by individuals with anxiety or depression [80]. It is also a key risk factor for negative mental and physical health outcomes, including increased risk of early mortality [44], [64], [6], [68], [8]. Despite its prevalence and adverse consequences, social disconnection shows only modest improvement following currently available psychosocial treatments for anxiety and depression [42], [78], suggesting these interventions do not sufficiently engage the mechanisms underpinning social connectedness.

### 1.2 Social disconnection and the bivalence model

Social disconnection represents a multifaceted construct encompassing deficits in functional, structural, and qualitative domains of social relationships [43]. This study focuses on two conceptually related but distinct *functional* indicators: *friendship* and *loneliness*. Friendship reflects perceived availability of companions for interaction or affiliation, whereas loneliness captures subjective feelings of social isolation [22].

Social contexts present people with opportunities for both reward (e.g., social relationship formation or strengthening) and punishment (e.g., rejection). One’s affective sensitivity in anticipation of or in response to these outcomes may therefore guide their experiences of social connection. Thus, it is theorized that social disconnection stems from under-activation of the positive valence system (PVS) and over-activation of the negative valence system (NVS) within social contexts [9], [50], [62], [61], [91], [95]. The PVS and its corresponding affective states (i.e., positive affect; PA) promote approach-oriented motivation and behaviors (e.g., initiating interactions, pursuing social opportunities) and heightened responsiveness to rewarding social experiences [33], [32], [38], [53], [63], [76]. In contrast, the NVS and its corresponding affective states (i.e., negative affect; NA) regulate avoidance-oriented motivation and behaviors (e.g., social withdrawal, reduced social engagement) and threat processing (e.g., exaggerated cognitive and affective sensitivity to social threat/rejection, including a heightened tendency to anxiously expect, readily perceive, and strongly react to rejection; [37], [46], [99]).

Cross-sectional research in non-clinical samples largely supports the bivalence model, linking greater social disconnection with elevated NA and reduced PA – patterns observed in both self-report and neural measures of emotional processing [62], [61], [90]. Although less common, longitudinal studies in non-clinical populations suggest loneliness predicts subsequent increases in NA – specifically, depressive symptoms – based on assessments conducted months to years apart [10], [58]. In one ecological momentary assessment study using short-term intervals (twice daily over two weeks), higher NA – operationalized as stress – predicted subsequent increases in loneliness in undergraduate students [104]. The temporal dynamics between PA and social disconnection remain understudied.

### 1.3 Applying the bivalence model of social disconnection to anxiety and depression

Anxiety and depressive disorders are marked by *attenuated PA* (e.g., anhedonia, or the reduced capacity and motivation to experience pleasure or reward [3], [15], [18], [49], [84], [92] and *heightened NA* (e.g., dysphoria, or persistent low mood, distress, anxiety, or irritability [101], [47], [7], [89]). Aligned with the bivalence model of social disconnection, cross-sectional research in clinical anxiety and depression samples shows greater social disconnection is related to lower PA and elevated NA [103], [85], [86], [95]. Further, ecological momentary assessment research and laboratory studies using social affiliation paradigms demonstrated that higher PA predicted greater perceived connectedness, lower loneliness, more social interactions, increased social approach behaviors, and greater desire for future interactions. Conversely, greater NA was related to higher loneliness, greater engagement in anxious and safety behaviors, and lower motivation for future social engagement [39], [77], [94].

Longitudinal research on affect and social disconnection in anxiety and depression is limited. Two multi-year studies of middle-aged and older adults with major depression found higher loneliness predicted greater subsequent NA — reflected in increased depression severity or higher risk of episode recurrence — based on assessments conducted 1–2 years apart [35], [45]. However, there is a lack of studies with shorter assessment intervals, such as weeks or months, that may better capture dynamic, proximal associations between affect and social disconnection. Examining these processes is particularly relevant for understanding treatment-related changes, as shifts in affect and social functioning may evolve over brief timescales.

Only one study to the authors’ knowledge [96] has examined the relationship between improvements in affect and changes in social connectedness throughout treatment for anxiety and depression. Taylor and colleagues demonstrated that increases in PA and to a lesser extent, decreases in NA (marginal effect), were independently associated with subsequent improvements in connectedness. However, elevated NA during treatment attenuated the association between PA and connectedness. Moreover, while greater connectedness predicted subsequent increases in PA, it did not significantly predict NA. Although informative, this study was limited by a small sample size and lack of control for autoregressive effects, which may introduce bias [1].

### 1.4 PVS-targeted interventions

Psychotherapy for anxiety [41] and depressive [26] disorders yield large reductions in NA, yet comparatively small improvements in PA. Responsive to this gap, researchers have developed positive valence-targeted interventions to enhance positive thoughts, behaviors, and emotions (e.g., [17], [19], [27], [56], [93]). These treatments, such as Amplification of Positivity (AMP; [93], [96]), effectively increase PA and reduce NA and anxiety and depression symptoms in clinical populations. Notably, AMP also demonstrates increases in self-reported social connectedness, neural indices of social reward response, and social approach behaviors during dyadic interactions [97], [93], [95]. Despite these promising findings, understanding *how* affective and social processes change throughout treatment remains limited.

## 2 Current study

### 2.1 Objectives and approach

The present study builds on prior work by examining whether PA and NA levels predict subsequent feelings of social connectedness (i.e., loneliness and friendship) throughout treatment, and whether social connectedness, in turn, predicts subsequent affect, in two independent AMP study samples using a dynamic longitudinal statistical modeling approach. Friendship and loneliness were analyzed as separate facets of social connectedness in the present study based on theoretical models [2] and empirical research demonstrating that, although related, they are non-redundant constructs with distinct correlates and developmental trajectories [75], [81], [82], [83], [88]. We investigated the within-person, time-lagged relationships between PA and NA and subsequent (a) loneliness and (b) friendship (Aim 1), and between loneliness and friendship and subsequent (a) PA and (b) NA throughout treatment (Aim 2), measured on a weekly or bi-weekly basis. An exploratory aim examined whether similar patterns emerged when substituting NA with anxiety and depressive symptoms. Longitudinal cross-lagged multilevel models with autoregression were conducted, consistent with statistical approaches implemented in recent research examining processes of change throughout mental health interventions [30], [29], [4], [79].

### 2.2 Primary aim hypotheses

#### 2.2.1 Predicting friendship

a. Higher PA will predict *greater* subsequent perceived friendship, beyond NA (**1a**).
b. Higher NA will predict *reduced* perceived friendship, beyond PA (**1b**).
c. An exploratory analysis will examine whether PA and NA interact to predict subsequent friendship.

#### 2.2.2 Predicting loneliness

a. Higher PA will predict *reduced* subsequent loneliness, beyond NA (**2a**).
b. Higher NA will predict *greater* subsequent loneliness, beyond PA (**2b**).
c. An exploratory analysis will examine whether PA and NA interact to predict subsequent loneliness.

### 2.3 Secondary aim hypotheses

#### 2.3.1 Predicting PA

a. Greater perceived friendship will predict *higher* subsequent PA, beyond loneliness (**3a**).
b. Greater loneliness will predict *reduced* subsequent PA, beyond perceived friendship (**3b**).

#### 2.3.2 Predicting NA

a. Greater perceived friendship will predict *reduced* subsequent NA, beyond loneliness (**4a**).
b. Greater loneliness will predict *higher* subsequent NA, beyond perceived friendship (**4b**).

***Exploratory Aim:*** An exploratory aim sought to re-estimate the models specified in the primary and secondary aims, substituting NA with measures of anxiety and depressive symptoms.

## 3 Method

### 3.1 Participants

Participants from both clinical trials (*N* = 98) were recruited from the general community; this analysis includes those randomized to receive AMP between April 2018 and July 2019 (*N* = 43; [97]; NCT03196544) and March 2021 and September 2023 (*N* = 55; NCT04945239). The sample included individuals ages 18–55 who endorsed clinically impairing anxiety (OASIS ≥ 8; [13], [71]) or depression (PHQ-9 ≥ 10; [54]), social disconnection (SCS-R < 90; [57]), and social functioning impairments (SDS-Social ≥ 5; [59]). Full eligibility criteria and symptom measures for study inclusion, identical across trials, are provided in the Supplement.

### 3.2 Measures

#### 3.2.1 Weekly assessments throughout treatment

**Positive and negative affect.** The Positive and Negative Affect Schedule (PANAS; [102]) is a 20-item self-report measure of affective states. It includes two 10-item subscales assessing high-arousal positive (e.g., interested, excited) and negative emotions (e.g., afraid, upset). Participants rate the extent to which they experienced each emotion during the past week on a 1 (*very slightly*) to 5 (*extremely*) Likert scale. The PANAS demonstrates strong internal consistency [20], [40].

**Social connectedness**. The NIH Toolbox Friendship and Loneliness Surveys assessed two facets of social connectedness: perceived friendship and loneliness [22]. For each measure, participants rate how much each statement applies on a scale from 1 (*never*) to 5 (*always*). The Friendship Survey includes eight items (e.g., “There are people around with whom to have fun;” “I feel close to my friends”); scores range from 8 to 40, with higher scores reflecting greater perceived availability of friends for interaction. The Loneliness Survey includes five items (e.g., “I feel lonely;” “I feel left out”); scores range from 5 to 25, with higher scores indicating greater feelings of social isolation and loneliness. Both measures demonstrate strong validity and internal reliability [22]. Consistent with prior literature, the median inter-correlation between friendship and loneliness across repeated assessments in the current study was *r* = -.58 (*mean r* = -.50), indicating related but distinct constructs [16], [72].

**Anxiety and depression symptoms.** The PROMIS Emotional Distress-Depression-Short Form (PROMIS-D-SF; [14]) is an 8-item measure of depressive symptom severity assessing general feelings of depression (e.g., worthless, helplessness, hopelessness) over the past week. The PROMIS Emotional Distress-Anxiety-Short Form (PROMIS-A-SF; [14]) is a 7-item measure of anxiety symptom severity assessing general feelings of anxiety (e.g., fearful, anxious, worried) over the past week. Responses on both scales are rated on a 5-point scale from 1 (*never*) to 5 (*always*), with higher total scores indicating greater symptom severity.

### 3.3 Amplification of Positivity treatment

Amplification of Positivity (AMP) is a structured, clinician-guided positive valence-targeted psychosocial intervention comprising three core components: (1) enhancing awareness of and responsiveness to positive events, (2) cultivating gratitude, and (3) engaging in acts of kindness. The 5- and 10-session AMP doses (Trial 1) and 6-session AMP dose (Trial 2) all included exercises targeting these components. Supplemental Table 1 provides a breakdown of the AMP modules administered across doses and trials.

### 3.4 Design and procedure

Study procedures were approved by the University of California San Diego Institutional Review Board. All participants provided written informed consent prior to participation. For both trials, eligible participants completed baseline assessments, including self-report measures, behavioral tasks, and neuroimaging. In the first trial, participants were randomly assigned to one of two doses of AMP (5 or 10 sessions) or the waitlist. In the second trial, participants were randomly assigned to either 6 sessions of AMP or stress management training. All participants randomized to AMP were included in the current analyses.

Participants completed self-report measures of positive and negative affect (PANAS), social connectedness (NIH Friendship and Loneliness surveys), and anxiety and depression (PROMIS-D-SF; PROMIS-A-SF) at pre-treatment, throughout the course of treatment, and at post-treatment. Assessment schedules varied across trials and arms, with some conducted remotely and others immediately before sessions; Supplemental Table 2 outlines the schedule for each trial.

### 3.5 Data analytic plan

Statistical analyses were performed in RStudio (https://www.r-project.org/). Data from the 5-, 6-, and 10-session treatments were pooled for the present analyses. Sensitivity analyses adjusting for trial (i.e., Trial 1 or 2) and dosage (5-, 6-, or 10-session AMP protocol) did not influence outcomes (see footnotes). Attrition was approximately 15 % in Trial 1 (5- and 10-session doses of AMP) and 18 % in Trial 2 (6-session dose of AMP). Multilevel modeling (MLM) was conducted using the "nlme" package to run longitudinal cross-lagged panel analyses across weekly assessments throughout treatment. MLMs were applied using an intent-to-treat approach, including all randomized participants, and adjusting for within-person clustering [87]. This analytical method aligns with previous research addressing similar questions [4]. In all models, the predictor at the prior time point was treated as a time-varying predictor of the outcome at the next assessment. Restricted maximum likelihood (REML) estimation was employed to account for missing data (including due to attrition) in the analyses.^1^

MLM is well-suited for analyzing longitudinal data with unequally spaced observations and unbalanced designs [65]. This flexibility is particularly advantageous when participants vary in the number or timing of assessments, as is common when combining data across similar, but not identical, trials. By nesting repeated observations *within* individuals, MLM inherently accounts for individual differences in assessment timing (as well as other person-specific characteristics). Thus, provided that time intervals are relatively consistent within individuals (to determine more stable *patterns* of associations) and random slopes are included in each model (to account for individual variability in change), incorporating data from both weekly and biweekly assessments (as occurred across the two trials for the current study) can improve generalizability and statistical power without compromising the validity of inferences.

**Primary Aim.** The following cross-lagged relationships were examined to address the primary aims of this study: PA and NA at time “t” as predictors of (1) friendship and (2) loneliness at time “t + 1.” See Supplemental Figure 1 for an example of the within-person analysis component. An interaction term between within-person PA and NA was added to each model to explore whether the effects of PA on subsequent friendship and loneliness, respectively, were attenuated at high levels of NA, and vice versa. Main-effects models were retained for interpretation in cases where the interaction was non-significant.

**Secondary Aim.** To address this study’s secondary aims, the reversed cross-lagged relationships were examined; Specifically: friendship and loneliness at time “t” as predictors of (3) PA at time and (4) NA at time “t + 1”.

**Exploratory Aim.** For exploratory analyses, anxiety and depression symptoms replaced NA in each model to enhance clinical relevance and better capture symptom-based components of NA. The models were as follows: PA, anxiety, and depression at time “t” as predictors of (5) friendship and (6) loneliness at time “t + 1.” We then examined whether friendship, loneliness, and PA at time “t” predicted (7) anxiety and (8) depression at time “t + 1”.

Predictors were disaggregated into between-persons components (each individual's mean score across all assessment time points) and within-person deviations (the difference between each time point and the individual's mean), reflecting change over time [100]. Given the focus on within-person change throughout treatment and the potential for between-person effects to be confounded by third variables, this study examined cross-lagged paths from the *within-person* component of each predictor to the outcome at the next time point (consistent with prior work, e.g., [4]).

All cross-lagged panel models included autoregressive effects by modeling each outcome at time "t" as a predictor of the same outcome at time “t + 1.” An autoregressive error structure was used to account for covariance among repeated measures over time. A random intercept captured individual differences in pre-treatment (baseline) levels of the outcome variables. Baseline scores were also included as covariates in all models to control for initial differences in the dependent variables.

For models in which PA was the predictor of interest, NA was included as a simultaneous cross-lagged predictor of the outcome (i.e., loneliness or friendship), and vice versa. This approach allowed estimation of each predictor’s unique effect while controlling for other. Similarly, when friendship was the predictor of interest, loneliness was included as a simultaneous cross-lagged predictor of the outcome (i.e., PA or NA), and vice versa. To ensure that including both predictors in the same model did not introduce problematic multicollinearity, variance inflation factors (VIFs) were examined. VIF values greater than 5 suggest moderate multicollinearity, whereas values greater than 10 indicate serious concerns (e.g., [55], [73]). All VIF values were below 5. All predictors were treated as disaggregated, time-varying predictors. To account for time-related changes, a “wave” covariate was included, which represented the treatment week. This allowed us to confirm findings were not simply a function of change over *time*.

To address multiple comparisons across the cross-lagged paths, we applied the Benjamini–Hochberg false discovery rate (FDR) adjustment [5]. Adjusted *p*-values are reported as *“q”* values in the results.

## 4 Results

Table 1 presents demographic and clinical characteristics of the current sample. Independent samples *t*-tests and chi-squared tests showed no significant differences between the two clinical trial samples on any baseline characteristics (i.e., age, OASIS, PHQ-9, SDS, SCSR, gender, ethnicity, race [White vs. non-White], PANAS subscales, Loneliness, Friendship, PROMIS anxiety and depression symptoms; all *p*s > .05). Correlations between baseline PA, NA, loneliness, friendship, anxiety, and depression are reported in Supplemental Table 3. Supplemental Table 4 summarizes descriptive statistics for variables of interest and symptom levels at each timepoint, calculated from the raw data. MLM results are provided below. Table 2, Table 3, Table 4, Table 5 present full results from the cross-lagged MLMs.

**Table 1:** Sociodemographic and Clinical Characteristics of Participants at Baseline.

**Variable**	
	***M (SD)***
Age (18–55 years)	30.43 (10.24)
OASIS	11.27 (2.61)
PHQ−9	14.03 (4.72)
SDS	19 (5.03)
SCSR	59.38 (14.14)
Positive Affect	20.66 (6.37)
Negative Affect	28.19 (6.58)
Loneliness	16.87 (3.57)
Friendship	21.68 (6.36)
Anxiety (PROMIS)	24.66 (4.42)
Depression (PROMIS)	25.86 (6.81)
	***n (%)***
**Gender**	
Female	31 (31.6)
Male	66 (67.3)
Other	1 (1.01)
**Ethnicity**	
Hispanic	23 (23.5)
Non-Hispanic	75 (76.5)
**Race**	
Asian American	28 (28.6)
Multiracial	8 (8.16)
Black / African American	2 (2.04)
Native American	0 (0)
Native Hawaiian/Pacific Islander	1 (1.02)
Middle Eastern/North African	2 (2.04)
White	49 (50)
Unknown/Declined to Respond	8 (8.16)
**Primary Diagnosis**	
MDD	37 (37.8)
SAD	19 (19.4)
GAD	31 (31.6)
PTSD	2 (2.04)
OCD	2 (2.04)
Other or Not Reported	7 (7.14)

*Note*. OASIS = Overall Anxiety Severity and Impairment Scale; PHQ-9 = Patient Health Questionnaire; SDS = Sheehan Disability Scale; SCSR = Social Connectedness Scale Revised; Positive and negative affect were measured using the PANAS (Positive and Negative Affect Schedule); Loneliness and friendship were measured using the NIH Toolbox Friendship and Loneliness Surveys; Anxiety and Depression were measured using the PROMIS Emotional Distress-Anxiety-Short Form and PROMIS Emotional Distress-Depression-Short Form, respectively. MDD = major depressive disorder; SAD = social anxiety disorder; GAD = generalized anxiety disorder; PTSD = post-traumatic stress disorder; OCD = obsessive-compulsive disorder.

**Table 2:** Cross-lagged MLM with Autoregression: Affect Predicting Subsequent Friendship.

**Predictor**	**Unstandardized Coefficient (*****b*****)**	**Std Error**	**DF**	**t-value**	**p-value**	**Standardized*****β***	**95 % CI Lower**	**95 % CI Upper**
**Intercept**	−1.6073	0.8182	335	−1.9644	0.0503	0.0000	−0.0284	0.0284
**Time**	0.2199***	0.0448	335	4.9045	< .001	0.0834	0.050	0.117
**PA**_**CWC**_	0.0744**	0.0261	335	2.8493	0.0047	0.0517	0.016	0.0874
**PA**_**mean**_	0.0052	0.0217	91	0.2399	0.8109	0.00433	−0.0315	0.0402
**NA**_**CWC**_	−0.0203	0.0264	335	−0.7680	0.4430	−0.0136	−0.0484	0.0212
**NA**_**mean**_	0.0129	0.0209	91	0.6169	0.5388	0.00984	−0.0218	0.0415
**Friendship**_**CWC**_	0.2389***	0.0470	335	5.0858	< .001	0.0886	0.0543	0.123
**Friendship**_**mean**_	1.1179***	0.0414	91	26.9744	< .001	1.0400	0.962	1.12
**Baseline Friendship**	−0.1107**	0.0397	91	−2.7910	0.0064	−0.0953	−0.163	−0.0275

*Note*. Outcome variable was perceived friendship at time *t* + 1*. CWC* subscript = centered within cluster lagged predictor, indicating the within-person deviation in predictor at prior time point; *mean* subscript = person-mean lagged predictor (indicating the between-person effect); PA = positive affect at time *t*; NA = negative affect at time *t*; Friendship = perceived friendship at time *t* (to account for autoregressive effects); Baseline Friendship = perceptions of friendship at baseline (pre-treatment). * *p* < .05; ** *p* < .01; *** *p* < .001.

**Table 3:** Cross-lagged MLM with Autoregression: Affect Predicting Subsequent Loneliness.

**Predictor**	**Unstandardized Coefficient (*****b*****)**	**Std Error**	**DF**	**t-value**	**p-value**	**Standardized*****β***	**95 % CI Lower**	**95 % CI Upper**
**Intercept**	1.4214*	0.6885	335	2.0646	0.0397	0.0000	−0.0361	0.0361
**Time**	−0.2491***	0.0356	335	−7.0063	< .0001	−0.158	−0.2030	−0.1140
**PA**_**CWC**_	−0.0580**	0.0198	335	−2.9323	0.0036	−0.0676	−0.1130	−0.0222
**PA**_**mean**_	−0.0112	0.0155	91	−0.7253	0.4702	−0.0157	−0.0587	0.0273
**NA**_**CWC**_	0.0380	0.0201	335	1.8903	0.0596	0.0427	−0.0017	0.0871
**NA**_**mean**_	−0.0055	0.0187	91	−0.2963	0.7677	−0.0071	−0.0545	0.0403
**Loneliness**_**CWC**_	0.1603**	0.0506	335	3.1690	0.0017	0.0761	0.0289	0.1230
**Loneliness**_**mean**_	1.1054***	0.0489	91	22.6217	< .0001	0.9570	0.8730	1.0400
**Baseline Loneliness**	−0.0968*	0.0428	91	−2.2618	0.0261	−0.0805	−0.1510	−0.0098

*Note*. Outcome variable was loneliness at time *t* + 1*. CWC* subscript = centered within cluster lagged predictor, indicating the within-person deviation in predictor at prior time point; *mean* subscript = person-mean lagged predictor (indicating the between-person effect); PA = positive affect at time *t*; NA = negative affect at time *t*; Loneliness = feelings of loneliness at time *t* (to account for autoregressive effects); Baseline Loneliness = feelings of loneliness at baseline (pre-treatment). * *p* < .05; ** *p* < .01; *** *p* < .001.

**Table 4:** Cross-lagged MLM with Autoregression: Connectedness Predicting Subsequent Positive Affect.

**Predictor**	**Unstandardized Coefficient (*****b*****)**	**Std Error**	**DF**	**t-value**	**p-value**	**Standardized*****β***	**95 % CI Lower**	**95 % CI Upper**
**Intercept**	−1.4403	2.5123	333	−0.5733	0.5668	0.0000	−0.0530	0.0530
**Time**	0.4560***	0.0918	333	4.9646	0.0000	0.1620	0.0979	0.2260
**Friendship**_**CWC**_	0.2084*	0.0983	333	2.1198	0.0348	0.0727	0.0052	0.1400
**Friendship**_**mean**_	−0.0098	0.0536	91	−0.1837	0.8547	−0.0086	−0.1020	0.0848
**Loneliness**_**CWC**_	−0.0104	0.1377	333	−0.0755	0.9398	−0.0028	−0.0751	0.0696
**Loneliness**_**mean**_	−0.0048	0.0884	91	−0.0545	0.9567	−0.0024	−0.0879	0.0833
**PA**_**CWC**_	0.1622**	0.0503	333	3.2236	0.0014	0.1070	0.0416	0.1720
**PA**_**mean**_	1.0828***	0.0563	91	19.2420	0.0000	0.8540	0.7660	0.9420
**Baseline PA**	−0.0995*	0.0462	91	−2.1553	0.0338	−0.0806	−0.1550	−0.0063

*Note*. Outcome variable was positive affect at time *t* + 1*. CWC* subscript = centered within cluster lagged predictor, indicating the within-person deviation in predictor at prior time point; *mean* subscript = person-mean lagged predictor (indicating the between-person effect); Friendship = perceived friendship at time *t;* Loneliness = feelings of loneliness at time *t*; PA = positive affect at time *t* (to account for autoregressive effects); Baseline PA = positive affect levels at baseline (pre-treatment). * *p* < .05; ** *p* < .01; *** *p* < .001.

**Table 5:** Cross-lagged MLM with Autoregression: Connectedness Predicting Subsequent Negative Affect.

**Predictor**	**Unstandardized Coefficient (*****b*****)**	**Std Error**	**DF**	**t-value**	**p-value**	**Standardized*****β***	**95 % CI Lower**	**95 % CI Upper**
**Intercept**	0.5963	2.2975	333	0.2595	0.7954	−0.0000	−0.0541	0.0541
**Time**	−0.3085***	0.0887	333	−3.4775	0.0006	−0.1180	−0.1840	−0.0511
**Friendship**_**CWC**_	−0.0485	0.0925	333	−0.5247	0.6001	−0.0182	−0.0863	0.0499
**Friendship**_**mean**_	0.0096	0.0476	91	0.2005	0.8415	0.0090	−0.0801	0.0980
**Loneliness**_**CWC**_	0.1020	0.1298	333	0.7860	0.4324	0.0292	−0.0439	0.1020
**Loneliness**_**mean**_	0.0373	0.1024	91	0.3639	0.7168	0.0195	−0.0869	0.1260
**NA**_**CWC**_	0.1518**	0.0482	333	3.1504	0.0018	0.1030	0.0388	0.1680
**NA**_**mean**_	1.0984***	0.0602	91	18.2592	< .0001	0.8470	0.7550	0.9390
**Baseline NA**	−0.1091*	0.0438	91	−2.4924	0.0145	−0.0939	−0.1690	−0.0191

*Note*. Outcome variable was negative affect at time *t* + 1*. CWC* subscript = centered within cluster lagged predictor, indicating the within-person deviation in predictor at prior time point; *mean* subscript = person-mean lagged predictor (indicating the between-person effect); Friendship = perceived friendship at time *t;* Loneliness = feelings of loneliness at time *t*; NA = negative affect at time *t* (to account for autoregressive effects); Baseline NA = negative affect levels at baseline (pre-treatment). * *p* < .05; ** *p* < .01; *** *p* < .001.

### 4.1 Primary aim

#### 4.1.1 Affect predicting subsequent friendship

***Positive affect.*** MLM results indicated higher PA predicted greater subsequent perceived friendship, even after accounting for prior friendship and NA levels (*b* =.074, *SE* =.026, *β* =.052, *t*(335) = 2.85*, p* = .0047, *q* = .0105).^2^

***Negative affect.*** NA did not predict subsequent perceived friendship, controlling for prior friendship and PA levels (*b* = −.020, *SE* =.026, *β* = −.014, *t*(335) = -.77*, p* = .4430; *q* = .5696).^3^

#### 4.1.2 Affect predicting subsequent loneliness

***Positive affect.*** Higher PA predicted lower subsequent loneliness, even after accounting for prior loneliness and NA levels (*b* = −.058, SE =.020, *β* = −.068, *t*(335) = -2.93, *p* = .0036, *q* = .0081).^4^

***Negative affect.*** NA did not predict subsequent loneliness, controlling for prior loneliness and PA levels (*b* =.038, *SE* =.020, *β* =.043, *t*(335) = 1.89, *p* = .0596, *q* = .0766).^5^

#### 4.1.3 Exploring PA and NA interaction

PA and NA did not significantly interact in predicting subsequent feelings of friendship (*p* = .1050) or loneliness (*p* = .2044).

### 4.2 Secondary aim

#### 4.2.1 Connectedness predicting subsequent PA

***Friendship*****.** Greater perceived friendship predicted higher subsequent PA, even after accounting for prior loneliness and PA levels (*b* =.208, *SE* =.098, *β* =.073, *t*(333) = 2.12*, p* = .0348).^6^ This relationship was marginally significant after applying the FDR adjustment (*q* =.0626).

***Loneliness*****.** Loneliness did not predict subsequent PA, controlling for prior friendship and PA levels (*b* = −.010, *SE* =.138, *β* = −.003, *t*(333) = -.076*, p* = .9398, *q* = .9567).

#### 4.2.2 Connectedness predicting subsequent NA

***Friendship.*** Perceived friendship did not predict subsequent NA, controlling for prior loneliness and NA levels (*b* = −.049, *SE* =.093, *β* = −.018, *t*(333) = -.52*, p* = .6001, *q* = .8415).

***Loneliness.*** Loneliness did not predict subsequent NA, controlling for prior friendship and NA levels (*b* =.102, *SE* =.130, *β* =.029, *t*(333) = .786*, p* = .4324, *q* = .7784).

#### 4.2.3 Exploratory aim: substituting NA with anxiety and depression symptoms

Exploratory models substituting anxiety and depression symptoms for NA showed identical non-significant patterns, except that higher anxiety (controlling for PA) predicted greater subsequent loneliness (*b* =.077, *SE* =.036, *β* =.066, *t*(238) = 2.15*, p* = .0323),^7^ a finding that was marginally significant after the FDR correction (*q* =.0593). Full results from the symptom models are provided in the Supplement.

## 5 Discussion

This study examined the within-person, time-dependent relationships between affect (PA and NA) and social connectedness (loneliness and friendship) throughout Amplification of Positivity (AMP) treatment for anxiety or depression, using weekly or bi-weekly assessments. An exploratory aim investigated whether substituting NA with its symptom-based components – anxiety and depression – yielded similar results. Higher PA predicted greater subsequent perceived friendship and lower subsequent loneliness throughout treatment, even after accounting for the influence of NA or anxiety and depression symptoms. Greater perceived friendship predicted higher subsequent PA (but not NA), whereas loneliness did not predict subsequent affect. NA did not predict subsequent friendship or loneliness. Similarly, symptom levels were unrelated to connectedness, except that higher anxiety predicted greater subsequent loneliness. Effect sizes were generally small, as expected in within-person longitudinal designs with autoregression, in which standardized effects capture subtle fluctuations around an individual’s mean (cf. between-person differences). Thus, small effects may nonetheless represent meaningful dynamic processes [1], [100], [21].

### 5.1 Primary aim

#### 5.1.1 Affect predicting subsequent social connectedness

In support of our hypotheses (1a and 2a), higher PA predicted greater subsequent friendship and lower subsequent loneliness throughout treatment, with small-sized effects. These findings align with prior research [96] and broader evidence linking greater PA to social connectedness in anxiety or depression (e.g., [25], [39]). According to the broaden-and-build theory [32], PA may enhance social functioning by expanding one’s thought-action repertoire and promoting approach-oriented behaviors (e.g., social initiation, openness) which, in turn, support the formation and maintenance of social bonds, providing more opportunities for connection and building social capital over time (e.g., [36], [38]). PA may also contribute to connectedness through social signaling pathways, whereby visible expressions of PA (e.g., smiling) attract others and reinforce reciprocal social affiliation (e.g., [74]). This mechanism is consistent with evolutionary theories positing that emotions serve key social functions for survival, such as affiliation, cooperation, and group cohesion [51], [66], [67], [70].

Contrary to our hypotheses (1b and 2b), NA did not predict subsequent loneliness or friendship beyond PA. This finding aligns with prior experimental and treatment-based research showing weaker longitudinal associations between NA and connectedness, relative to PA, in individuals with anxiety or depression (e.g., [96], [94]). However, this finding contrasts with between-person studies linking higher NA to greater loneliness – most of which were cross-sectional, non-intervention-based, and conducted in non-clinical samples (e.g., [52]; see review by Luo & Shao, 2023). It is possible that cross-sectional associations reflect *concurrent* distress rather than directional effects over time. Timing of assessments may also help explain the null findings. For example, NA could have more immediate or short-lived effects on social perceptions, whereas PA may drive cumulative changes that build social connection over days or weeks, producing more sustained gains. Further, although AMP has been shown to meaningfully reduce NA [93], it is specifically designed to target the PVS. The emphasis on PA in psychoeducation, assignments, and in-session discussions may have heightened participants’ awareness of PA fluctuations (relative to NA) throughout treatment; however, this possibility requires empirical scrutiny. Current study findings suggest that NA is less directly involved in shaping subsequent social experiences compared to PA, which — when modeled simultaneously — accounted for more variance in social connectedness outcomes. This interpretation is supported by exploratory post-hoc analyses removing PA (see Footnotes 3 and 5 on page 15), which showed NA significantly predicted greater subsequent loneliness — but not friendship — when PA was excluded. This aligns with prior literature demonstrating positive associations between NA and loneliness (e.g., [12], [11], [10]; see review by Luo & Shao, 2023). However, the absence of this association when PA was included in the model underscores the importance of examining PA alongside NA when investigating mechanisms of social disconnection and possible treatment targets in anxiety and depression. The finding that NA predicted subsequent loneliness, but not friendship, reinforces conceptualizations of social connectedness as a multifaceted construct [43] and suggests that affective processes may differentially relate to distinct sub-components of social connectedness.

Exploratory analyses indicated PA and NA did not interact to predict subsequent friendship or loneliness. This contrasts with prior findings showing elevated NA blunted the impact of PA on later social connectedness throughout treatment, and vice versa (i.e., PA attenuated NA’s effect on connectedness; [96]). Differences in methodology (e.g., separate measures of friendship and loneliness vs. a single social connectedness scale), sample characteristics, or statistical approaches may account for this discrepancy. The null interactions may also reflect limited power, given the complexity of cross-lagged MLMs involving interaction terms. Notably, prior research in both experimental and naturalistic settings has yielded mixed findings regarding the interaction between PA and NA in predicting social connectedness, with some studies finding the interaction [23], [39], [96] and others finding no effect [95]. Further research is needed to clarify whether and how PA and NA interact during treatment, which could guide more personalized combinations of positive and negative valence-focused interventions and help identify who is most likely to benefit from each.

### 5.2 Secondary aim

#### 5.2.1 Social connectedness predicting subsequent affect

As hypothesized (3a), friendship predicted higher subsequent PA, supporting a positive bidirectional relationship between these constructs – although the effect was marginally significant following FDR correction. Friendship did not, however, predict subsequent NA (disconfirming hypothesis 4a). These findings align with previous work indicating connectedness is more strongly associated with later increases in PA than NA [96]. They are also broadly consistent with the Broaden-and-Build theory [31], [32], which proposes a reinforcing “upward spiral” wherein PA promotes social connection, which in turn supports further increases in PA [34], [76]. Conversely, although friendship may buffer negative affect to some extent, its impact might be too limited to produce significant changes in subsequent NA levels, which are likely influenced by other factors. For example, the significant reduction in NA following AMP treatment (e.g., [93]) may reflect multiple mechanisms beyond friendship or loneliness, including other social factors like perceived support and non-social factors such as improved self-efficacy and emotion regulation skills that may be developed through treatment. In contrast to our expectations (hypotheses 3b and 4b), changes in loneliness did not significantly predict subsequent PA or NA, suggesting that reductions in loneliness may not directly translate to affective improvements – at least within the timescales examined here. One possibility is that the PVS-focused strategies participants engaged with during AMP buffered the emotional impact of loneliness over time. The null association between loneliness and subsequent NA is consistent with prior longitudinal research showing similar patterns (e.g., [24], [48], [98], possibly indicating that loneliness may reflect a concurrent symptom of NA rather than a causal antecedent. Likewise, the absence of a link between loneliness and PA supports the idea that positive and negative valence systems may function independently (e.g., [69], [77]), and that increases in PA may stem not from the absence of negative states like loneliness, but from the presence of enriching experiences like friendship that help catalyze upward spirals of well-being [34], [76], [96].

### 5.3 Exploratory aim: symptom models

#### 5.3.1 PA, anxiety, and depression predicting subsequent social connectedness

When anxiety and depression were substituted for NA in models alongside PA predicting subsequent friendship, neither symptom variable significantly predicted changes in friendship. However, in models predicting subsequent loneliness, higher anxiety (but not depression) symptoms predicted increased loneliness at the next time point, an effect that was marginally significant following FDR correction. This pattern is consistent with prior research suggesting anxiety – particularly social anxiety – is closely associated with loneliness, largely due to heightened fears of rejection, negative self-perceptions, and avoidance of social interactions. In contrast, while depression is also linked to loneliness, its influence may stem more from social withdrawal and motivational deficits, reflecting PA deficits (i.e., anhedonia), rather than elevated NA [12], [28], [60], [88].

Notably, in all symptom models, higher PA remained a significant predictor of greater subsequent friendship and lower loneliness, even after accounting for anxiety and depression symptoms. This underscores the unique contribution of positive emotional experiences to social connection, beyond the effects of symptom reduction alone. These findings align with meta-analytic evidence indicating that while psychotherapy often yields substantial improvements in anxiety and depression, gains in social functioning and connectedness are comparatively modest [42], [78]. Even after achieving symptom remission, many individuals continue to experience persistent social disconnection, including loneliness and low perceived social support [80]. Taken together, these results emphasize the need for interventions that not only alleviate distress but also actively target PA to more comprehensively address social disconnection and functional impairment.

#### 5.3.2 Social connectedness and PA predicting subsequent anxiety and depression

Neither perceived friendship nor loneliness significantly predicted subsequent anxiety or depression symptoms. One possibility is that short-term fluctuations in social connectedness may not exert strong immediate effects on symptom levels, or that their influence is indirect - operating primarily through changes in affective experience, particularly PA, which significantly predicted both outcomes. It is also possible that the mental health impact of social connectedness becomes more evident over longer timescales than those captured in the current study. Alternatively, meaningful associations may unfold over even shorter time frames – such as hours or days – highlighting the value of ecological momentary assessment (EMA) approaches for detecting dynamic, within-person relationships that might be missed with weekly assessments. Future research leveraging both extended follow-up and fine-grained temporal designs may help clarify when and how social connection contributes to symptom change.

## 6 Limitations

This study should be interpreted in the context of several limitations. First, samples were combined from two separate AMP trials involving three distinct doses (i.e., number) of treatment sessions. Although sensitivity analyses controlling for these variables confirmed that the main findings were consistent, and no group differences were observed on demographics or key variables, unmeasured study- or condition-related differences may still have introduced potential confounds. Second, affect, social, and symptom measures were assessed using retrospective self-report. Although self-report is well-suited for capturing subjective experiences such as loneliness and perceived friendship, incorporating complementary methods, such as behavioral indicators or informant reports, could offer additional insight into social functioning. Third, assessments were administered weekly or biweekly in this study. Future studies may apply time series analyses to determine the optimal and most clinically meaningful time lags for this purpose. Fourth, all participants in this study endorsed impairments in social connection and functioning, which may limit generalizability to anxious or depressed samples without such pronounced social difficulties. Although elevated levels of social impairment are common among individuals with depression and anxiety (e.g., [80]), it is possible that the degree of impairment in this sample may have influenced associations between affective states and social connectedness. Future research should examine whether similar patterns emerge in anxious and depressed populations along the full distribution of social functioning impairments. Finally, replication in larger and more diverse samples, and across different types of interventions (e.g., those targeting the NVS), is needed to determine the generalizability of these findings.

## 7 Conclusion

The present findings highlight the predictive role of greater PA on subsequent improvements in social connectedness (i.e., loneliness and perceived friendship) throughout AMP treatment for anxiety or depression. Results suggest that interventions specifically targeting the PVS by upregulating PA (e.g., [19], [27], [56], [93], [97]) may offer particular benefits for reducing social disconnection in this population. Assessing where individuals fall on the PA spectrum may also help guide more personalized and effective treatment strategies that address affective processes contributing to social disconnection in anxiety and depression. For example, incorporating PA-enhancing strategies into standard NVS-focused treatments for anxiety and depression (e.g., cognitive behavioral therapy) may improve outcomes for some individuals by reducing loneliness and strengthening perceived friendship. Ongoing assessments of PA and social disconnection throughout treatment could help tailor care by identifying individuals who may benefit from more intensive PA-focused modules or targeted interventions. Future research should examine the most effective timing and format for these strategies — whether as standalone treatments, sequenced or fully integrated components, or supplemental tools for individuals with pronounced deficits in PA and/or social connectedness. It will also be important to identify which components of AMP or other PVS-targeted interventions most effectively reduce social disconnection to streamline treatment and optimize impact. Finally, interventions may benefit from explicitly leveraging the reciprocal relationship between PA and social connection to create a reinforcing cycle of emotional and social improvement — moving beyond symptom reduction toward more robust and lasting well-being.

## Funding

This research was supported by grants awarded to Charles T. Taylor from the 10.13039/100000025National Institute of Mental Health R61MH113769 and R33MH113769. The project described was partially supported by the 10.13039/100000002National Institutes of Health, Grant UL1TR000100 of CTSA funding prior to August 13, 2015 and Grant ULTR001442 of CTSA funding beginning August 13, 2015 and beyond.

## Declaration of Competing Interest

Charles T. Taylor declares that in the past 3 years he has been a paid consultant for Bionomics, atai Life Sciences, and Engrail Therapeutics, and receives payment for editorial work for *UpToDate. Murray B. Stein declares that in the past 3 years he has received consulting income from Acadia Pharmaceuticals, Aptinyx, atai Life Sciences, BigHealth, Biogen, Bionomics, BioXcel Therapeutics, Boehringer Ingelheim, Clexio, Eisai, EmpowerPharm, Engrail Therapeutics, Janssen, Jazz Pharmaceuticals, Newleos Therapeutics, PureTech Health, Sage Therapeutics, Seaport Therapeutics, Sumitomo Pharma, Roche/Genentech, and Transcend Therapeutics. Dr. Stein has stock options in Oxeia Biopharmaceuticals, EpiVario and Newleos. He has been paid for his editorial work on Depression and Anxiety (Editor-in-Chief), Biological Psychiatry (Deputy Editor), and UpToDate (Co-Editor-in-Chief for Psychiatry). He has also received research support from NIH, Department of Veterans Affairs, and the Department of Defense. He is on the scientific advisory board for the Brain and Behavior Research Foundation and the Anxiety and Depression Association of America. Madeleine M Rassaby and Samantha N Adams declare no conflicts of interest.*

All procedures performed involving human participants were in accordance with the ethical standards of the University of California San Diego Human Research Protection Program and with the Code of Ethics of the World Medical Association (Declaration of Helsinki).

## Footnotes

1 Participants who completed only baseline or one follow-up contributed to between-person estimates (e.g., intercepts), but not to within-person cross-lagged or autoregressive estimates, which require multiple observations. Most participants provided sufficient timepoints to inform dynamic within-person effects.

2 PA findings held when considering the influence of treatment dosage (five, six, or 10 sessions of AMP; *b* =.072, *SE* =.026, *β* =.050, *t*(335) = 2.73*, p* = .0066) and clinical trial (Trial 1 or Trial 2; *b* =.070, *SE* =.026, *β* =.049, *t*(335) = 2.70*, p* = .0074), respectively, as covariates in the prediction of subsequent friendship levels.

3 When the friendship prediction model was run with NA alone (excluding PA), the relationship remained non-significant (*b* = −.045, *SE* =.025, *β* = −.031, *t*(336) = -1.82*, p* = .0685, *q* = .0799)

4 PA findings held when considering the influence of treatment dosage (*b* = −.056, *SE* =.020, *β* = −.065, *t*(335) = -2.82*, p* = .0052) and clinical trial (*b* = −.055, *SE* =.020, *β* = −.064, *t*(335) = -2.79*, p* = .0056), respectively, as covariates in the prediction of subsequent loneliness.

5 Given this surprising finding, the loneliness prediction model was run with NA alone (excluding PA). Results indicated that higher NA predicted greater subsequent loneliness after controlling for prior loneliness (*b* =.058, *SE* =.019, *β* =.065, *t*(336) = 13.02*, p* = .0028, *q* = .0048)

6 Friendship findings held when considering the influence of treatment dosage (*b* =.210, *SE* =.090, *β* =.072, *t*(334) = 2.33*, p* = .0206) and clinical trial (*b* =.204, *SE* =.090, *β* =.071, *t*(334) = 2.26*, p* = .0243), respectively, as covariates in the prediction of subsequent PA.

7 Anxiety findings held when considering the influence of treatment dosage (*b* =.076, *SE* =.036, *β* =.064, *t*(238) = 2.11*, p* = .0356) and clinical trial (*b* =.076, *SE* =.036, *β* =.065, *t*(238) = 2.12*, p* = .0347), respectively, as covariates in the prediction of subsequent loneliness.
