# Supplementary material for: The power of positivity: Exploring affective and social processes of change throughout a positive valence treatment for anxiety or depression
Source: J Mood Anxiety Disord. 2025 Aug 27;12:100146. doi: 10.1016/j.xjmad.2025.100146 (PMC12452861; doi:10.1016/j.xjmad.2025.100146)
Supplement: Supplementary file 1 — Supplementary material [file mmc1.docx]

**Supplemental Materials**

**Detailed Inclusion / Exclusion Criteria**

*Note that the inclusion and exclusion criteria were identical across trials (NCT03196544 and NCT04945239)*

| **Inclusion Criteria** | **Exclusion Criteria** |
| --- | --- |
| - PHQ-9 ≥ 10 and/or OASIS ≥ 8  - Social Connectedness Scale Revised (SCSR) < 90  - Sheehan Disability Scale (SDS) - Social Domain ≥ 5  - Ages 18-55, inclusive  - Proficient in English language | - No telephone access  - Past year substance use disorder (exceptions: *mild* alcohol, nicotine, caffeine, and marijuana use disorders)  - Bipolar I or Psychotic disorders  - Moderate to severe TBI with neurological deficits, neurological disorders, or severe or unstable medical conditions  - Current regular use of anxiolytics, antipsychotics, antidepressants, mood stabilizers, beta-blockers, sleep medications, opioids/codeine, migraine medications.  - MRI contraindications  - Non-correctable vision or hearing problems  - Concurrent psychosocial treatment (or fail to meet 12-week therapy stability criteria)  - Inability to complete initial assessment battery or treatment sessions  - Clinical conditions that necessitate more imminent clinical care (e.g., active suicidal ideation) |

**Symptom Measures for Study Inclusion**

**Anxiety.** Anxiety symptoms were evaluated for study inclusion using the Overall Anxiety Severity and Impairment Scale (OASIS; Norman et al., 2006), a five-item self-report measure assessing the frequency and severity of anxiety, as well as associated avoidance behaviors and functional impairment over the past two weeks. Scores range from 0 to 20, with a cutoff of ≥ 8 indicating clinically significant anxiety (Campbell-Sills et al., 2009).

**Depression**. The Patient Health Questionnaire (PHQ-9) was administered to assess depressed mood (Kroenke et al., 2001). The 9-item self-report measure evaluates frequency of depressive symptoms (e.g., “feeling down, depressed, or hopeless”) over the past two weeks on a scale from 0 (*not at all*) to 3 (*every day*). Scores ≥ 10 indicate clinically significant depression (range: 0-27).

**Social Disconnection.** The Social Connectedness Scale Revised (SCS-R) was used to evaluate perceived social connectedness for study inclusion (Lee et al., 2001). This 20-item self-report measure captures feelings of belonging and connection within one’s social environment. Participants rate the extent to which various statements describe their self-perception on a scale from 1 (*strongly disagree*) to 6 (*strongly agree*). Examples of positively scored items include “I feel close to people” and “I fit in well in new situations,” and reverse-scored items include “I see myself as a loner” and “I don’t feel I participate with anyone or any group.” Higher total scores reflect greater perceived social connectedness.

**Social Functioning Impairments.** Social functioning impairments were assessed for study inclusion using the Sheehan Disability Scale-Social domain (SDS-Social; Leon et al., 1997). This measure asks participants to rate the extent to which their anxiety and/or depression symptoms have interfered with their social life or leisure activities, using a scale from 0 (*not at all*) to 10 (*extremely*). A score of 5 or higher indicates moderate to severe impairment.

**Supplemental Table 1**

*AMP Modules by Dose*

| **Session #** | **Trial I** | | **Trial II** |
| --- | --- | --- | --- |
|  | **5-Session Dose** | **10-Session Dose** | **6-Session Dose** |
| 1 | Psychoeducation | | |
| 1-2 | Responsiveness I (noticing and amplifying positive events) | | |
| 2 | Gratitude I (gratitude practice) | | |
| 3 | Prosocial Acts I (practicing acts of kindness) | | |
| 4 | Social Activity Scheduling | | |
| 5 | Review & Planning: Personalized positive activity plan | Responsiveness II  (active/constructive responding) | Combining Responsiveness, Gratitude, & Prosocial Acts |
| 6 | Weekly check-ins (by email or phone) to remind participants to practice personalized positive activity plan | Gratitude II  (expression) | Review & Planning: Personalized positive activity plan |
| 7 |  | Prosocial Acts II  (make someone else happier) |  |
| 8 |  | Responsiveness III  (live this month like it’s your last in this area) |  |
| 9 |  | Combining Responsiveness, Gratitude, & Prosocial Acts |  |
| 10 |  | Review & Planning: Personalized positive activity plan |  |

**Description of AMP Doses**

Across doses, sessions lasted one hour (with an additional 30 minutes for the first treatment session allotted to orientation and introductions) and followed a standard CBT structure, which included reviewing exercises from the prior week, problem-solving any challenges encountered, introducing a new concept and/or positive activity, and assigning between-session exercises. Across all treatment doses, sessions 1 to 4 shared the same initial four therapeutic strategies.

In Trial 1, the 10-session AMP format expanded upon this foundation by incorporating additional activities aligned with its core domains. In contrast, the 5-session AMP format involved a 30-minute follow-up phone call during week 6 to review participants’ individualized treatment plans, followed by weekly emails to support ongoing positive activity engagement, and optional phone check-ins from weeks 7 through 10. Sessions occurred weekly for both the 5- and 10-dose AMP interventions in Trial 1.

For the 6-session AMP format in Trial 2, sessions 1 to 4 occurred weekly, whereas sessions 5 and 6 occurred every other week, allowing for additional practice and consolidation of therapy skills. This dose of AMP aligned with the modules delivered in the 5-session dose, but included an expanded (60 minute) final session instead of the 30-minute phone call used in Trial 1.

**Supplemental Table 2**

*Assessment Schedule by Trial*

|  | **Trial 1** | | **Trial 2** |  |
| --- | --- | --- | --- | --- |
| **Timepoint** | **5-Session Dose** | **10-session Dose** | **6-session Dose** | **Combined Variable Key** |
| Pre-treatment | s1 | s1 | s1 | Wave 1 |
| Tx Week 1 | s2 | s2 | s2 | Wave 2 |
| Tx Week 2 | — | — | s3 | Wave 3 |
| Tx Week 3 | s3 | s3 | s4 | Wave 4 |
| Tx Week 4 | — | — | s5 | Wave 5 |
| Tx Week 5 | s5 | s5 | s5.5 | Wave 6 |
| Tx Week 6 | — | — | s6 | Wave 7 |
| Tx Week 7 | s7 | s7 | s6.5 | Wave 8 |
| Tx Week 8 | — | — | s7 | Wave 9 |
| Tx Week 9 | s9 | s9 | — | Wave 10 |
| Tx Week 10 | — | — | — | Wave 11 |
| Post-Treatment | s12 | s12 | s8 | Wave 12 |

*Note*. Tx = treatment; s = session.

**Supplemental Table 3**

*Correlation Matrix of Variables of Interest* *at Baseline*

| Variable | *M* | *SD* | **1** | **2** | **3** | **4.** | **5.** |
| --- | --- | --- | --- | --- | --- | --- | --- |
| **1. Positive Affect** | 20.66 | 6.37 | -- |  |  |  |  |
| **2. Negative Affect** | 28.19 | 6.58 | **-.12*** | -- |  |  |  |
| **3. Loneliness** | 16.87 | 3.57 | **-.27*** | **.32*** | -- |  |  |
| **4. Friendship** | 21.68 | 6.36 | **.22*** | **-.13*** | **-.64*** | -- |  |
| **5. Anxiety Symptoms** | 24.66 | 4.42 | **-.12*** | **.67*** | **.29*** | **-.13*** | -- |
| **6. Depression Symptoms** | 25.86 | 6.81 | **-.44*** | **.58*** | **.54*** | **-.30*** | **.42*** |

*Note*. * *p* < .01; All correlations are significant.

**Supplemental Table 4**

*Means and Standard Deviations of Variables of Interest Throughout AMP Treatment*

| **Assessment Timepoint** | **PA** | **NA** | **Loneliness** | **Friendship** | **Anxiety** | **Depression** |
| --- | --- | --- | --- | --- | --- | --- |
| **Baseline** | 20.7 (6.41) | 28.2 (6.61) | 16.9 (3.59) | 21.7 (6.39) | 24.7 (4.44) | 25.9 (6.84) |
| **Assessment 2** | 22.1 (6.71) | 24.9 (6.77) | 16.4 (3.79) | 21.9 (6.97) | 23.6 (4.13) | 24.6 (6.89) |
| **Assessment 3** | 25.1 (7.21) | 21.8 (6.27) | 15.4 (3.73) | 23.1 (7.09) | 21.3 (4.91) | 21.7 (6.61) |
| **Assessment 4** | 26.3 (7.69) | 22.8 (6.89) | 15.0 (3.97) | 23.3 (6.86) | 21.3 (4.91) | 22.4 (6.63) |
| **Assessment 5** | 25.7 (7.64) | 21.8 (7.30) | 14.8 (4.51) | 23.9 (7.58) | 20.9 (5.92) | 21.0 (6.75) |
| **Assessment 6** | 27.1 (8.88) | 21.8 (7.48) | 14.3 (4.29) | 24.2 (7.46) | 20.6 (5.48) | 20.8 (6.81) |
| **Assessment 7** | 26.8 (7.94) | 22.1 (7.81) | 13.8 (4.33) | 24.5 (7.97) | 20.7 (6.46) | 20.9 (7.07) |
| **Assessment 8** | 26.8 (7.94) | 20.5 (7.13) | 13.6 (4.04) | 24.0 (6.99) | 19.8 (5.48) | 20.5 (6.64) |
| **Assessment 9** | 28.0 (8.72) | 20.0 (7.13) | 13.2 (4.88) | 24.8 (8.25) | 19.8 (5.26) | 19.5 (7.06) |
| **Assessment 10** | 26.8 (9.35) | 20.6 (6.48) | 14.2 (3.69) | 23.7 (6.64) | 19.2 (4.19) | 20.4 (6.25) |
| **Post** | 28.1 (8.40) | 19.2 (5.80) | 12.8 (4.57) | 25.0 (7.58) | 18.5 (4.51) | 18.5 (6.92) |

*Note*. Values reported are *Mean (SD).*

**Supplemental Figure 1**

**
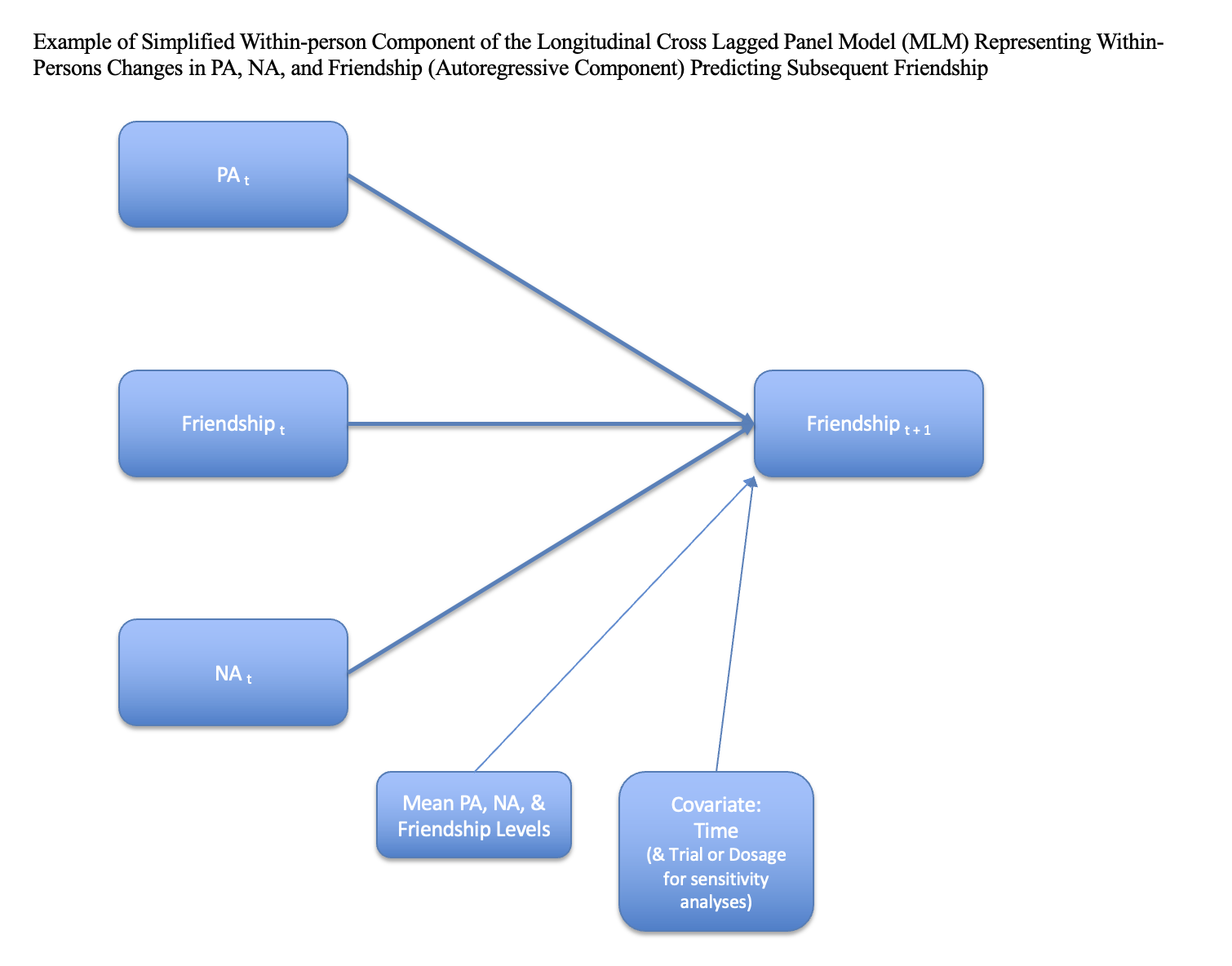
**

**Exploratory Aim Results: Substituting NA with Anxiety and Depression Symptoms**

***PA, Anxiety, and Depression Symptoms Predicting Subsequent Friendship***

***Positive Affect.*** MLM analyses indicated higher PA predicted greater feelings of friendship at the subsequent time point, controlling for prior anxiety and depression symptoms and friendship levels (*b* = .088, *SE* = .031, *β* = .065, *t*(238) = 2.86*, p* = .0047, *q* = .0130)^^[[1]](#footnote-1)^^.

***Anxiety and Depression Symptoms.*** Neither anxiety nor depression symptoms predicted feelings of friendship at the following time point, controlling for prior PA and friendship levels (*anxiety: b* = -.076, *SE* = .047, *β* = -.038, *t*(238) = -1.60*, p* = .1102, *q* = .2020; *depression:* *b* = .033, *SE* = .048, *β* = .018, *t*(238) = .68*, p* = .4942, *q* = .5489).

***PA, Anxiety, and Depression Symptoms Predicting Subsequent Loneliness***

***Positive Affect.*** Higher PA predicted lower loneliness at the subsequent time point, controlling for prior anxiety and depression symptoms and loneliness levels (*b* = -.051, *SE* = .023, *β* = -.063, *t*(238) = -2.19*, p* = .0292)^^[[2]](#footnote-2)^^. This relationship was marginally significant after applying the FDR adjustment (*q* = .0593).

***Anxiety and Depression Symptoms.*** Greater anxiety symptoms at one time point predicted higher loneliness at the following time point, controlling for prior depression symptoms, PA, and loneliness levels (*b* = .077, *SE* = .036, *β* = .066, *t*(238) = 2.15*, p* = .0323)^^[[3]](#footnote-3)^^, a relationship which was marginally significant after applying the Benjamini-Hochberg FDR adjustment (*q* = .0593). Depression symptoms did not significantly predict subsequent loneliness, controlling for prior anxiety, PA, and loneliness levels (*b* = -.050, *SE* = .037, *β* = -.046, *t*(238) = -1.34, *p* = .1815; FDR adjustment: *q* = .2496).

***Friendship and Loneliness Predicting Subsequent Anxiety***

***Friendship.*** Friendship did not predict subsequent anxiety symptoms, controlling for prior loneliness, PA, and anxiety (*b* = -.026, *SE* = .070, *β* = -.014, *t*(227) = -.37*, p* = .7095; FDR adjustment: *q* = .7805).

***Loneliness.*** Loneliness did not predict subsequent anxiety symptoms, controlling for prior PA and anxiety symptoms (*b* = -.116, *SE* = .105, *β* = -.047, *t*(227) = -1.11*, p* = .2703; FDR adjustment: *q* = .3303).

***Friendship and Loneliness Predicting Subsequent Depression***

***Friendship.*** Friendship did not predict subsequent depression symptoms, controlling for prior loneliness, PA, and depression symptoms (*b* = -.072, *SE* = .075, *β* = -.028, *t*(334) = -.952*, p* = .3416; FDR adjustment: *q* = .5368).

***Loneliness.***  Loneliness did not predict subsequent depression symptoms, controlling for prior PA and depression symptoms (*b* = .022, *SE* = .108, *β* = .006, *t*(334) = .200*, p* = .8414; FDR adjustment: *q* = .8414).

1. PA findings held when considering the influence of treatment dosage (*b* = .084, *SE* = .031, *β* = .062, *t*(238) = 2.71*, p* = .0071) and clinical trial (*b* = .082, *SE* = .031, *β* = .060, *t*(238) = 2.67*, p* = .0082), respectively, as covariates in the prediction of subsequent friendship. [↑](#footnote-ref-1)
2. PA findings held when considering the influence of treatment dosage (*b* = -.048, *SE* = .023, *β* = -.059, *t*(238) = -2.07*, p* = .0391) and clinical trial (*b* = -.047, *β* = -.058, *SE* = .023, *t*(238) = -2.04*, p* = .0424), respectively, as covariates in the prediction of subsequent loneliness. [↑](#footnote-ref-2)
3. Anxiety findings held when considering the influence of treatment dosage (*b* = .076, *SE* = .036, *β* = .064, *t*(238) = 2.11*, p* = .0356) and clinical trial (*b* = .076, *SE* = .036, *β* = .065, *t*(238) = 2.12*, p* = .0347), respectively, as covariates in the prediction of subsequent loneliness. [↑](#footnote-ref-3)
